# Supplementary material for: Emergence of electric-field-tunable interfacial ferromagnetism in 2D antiferromagnet heterostructures
Source: Nat Commun. 2022 Dec 15;13:7348. doi: 10.1038/s41467-022-34812-6 (PMC9755235; doi:10.1038/s41467-022-34812-6)
Supplement: Supplementary file 1 — Supplementary Information [file 41467_2022_34812_MOESM1_ESM.pdf]

Supplementary Information for

Emergence of electric-field-tunable interfacial ferromagnetism in 2D antiferromagnet heterostructures

Guanghui Cheng, Mohammad Mushfiqur Rahman, Zhiping He, Andres Llacsahuanga Allcca, Avinash Rustagi, Kirstine Aggerbeck Stampe, Yanglin Zhu, Shaohua Yan, Shangjie Tian, Zhiqiang Mao, Hechang Lei, Kenji Watanabe, Takashi Taniguchi, Pramey Upadhyaya, Yong P. Chen

Correspondence to: yongchen@purdue.edu

1. Discussion on other possible origins of the hysteretic Kerr rotation signal at low fields of the 2L CrI_3/FL $CrCl_3$

(a) *Symmetry-breaking effect in AFM.* The symmetry-breaking effect can lead to non-zero MOKE signal in antiferromagnetic materials [1], even with zero net magnetization. In principle, there's no MOKE signal for systems with either time reversal symmetry (*T*) or combined time reversal and inversion symmetry (*TI*)[2] (such as the pristine AFM bilayer CrI_3 with *TI* symmetry). An AFM material may become magneto-optically active when *TI* symmetry is broken by a perpendicular electric field [1] or substrate effect [3] or strain [4]. For example, it is reported that applying an electric field can induce a non-zero MOKE or magnetic circular dichroism (MCD) signal for the AFM phase of bilayer CrI_3 [5, 6]. However, in this case the hysteresis loop is qualitatively different from the one observed in our experiments. Namely, due to the lack of a net magnetization, a given layered AFM state (e.g., ↑↓) cannot be directly switched to its time reversed partner (e.g., ↓↑); instead, the time reversed state is reached via a spin-flip transition to a ferromagnetic state (e.g., ↑↑). Consequently, such symmetry breaking effect induces a finite MOKE signal in the AFM phase, and signal transition only occurs for the AFM-to-FM phase, giving rise to a hysteresis loop as schematically illustrated in Supplementary Fig. 6 and supported by the reported electric-field-

controlled magnetism in 2L CrI_3 [5]. In contrast, we observed an extra FM-type transition near zero field in addition to the high field induced spin-flip transitions for the 2L CrI_3/FL $CrCl_3$ heterostructures (Supplementary Fig. 6 and Fig. 1d), which is consistent with our proposed scenario where at least three spin layers (two layers of CrI_3 plus one neighboring layer of $CrCl_3$) are responsible for the observations, and is qualitatively different from the symmetry-breaking induced effect (Supplementary Fig. 6).

(**b**) *Bubble-induced strain.* Bubbles generally could exist in 2D heterostructures and the bubble-induced local strain may modulate the magnetism [4]. The AFM images of typical heterostructures suggest uniform interfaces with few bubbles (Supplementary Fig. 7a,b). During the MOKE measurement, we also intentionally parked the laser spot away from the visible deformations (e.g., bubbles, wrinkles) under the microscope (Supplementary Fig. 7c). In contrast to the random distribution of the bubbles, the emergent FM loop is well reproduced in almost all the heterostructure samples (summarized in Supplementary Fig. 8).

Moreover, bubbles could also exist in other 2D stacks, but we have never observed anomalous features (e.g., the FM loop) in BN/2L CrI_3/BN, including 2L CrI_3 regions in the heterostructure samples or pure 2L CrI_3 samples. Even if bubble-induced strain plays a role, the strain is reported to just modulate the magnetic interactions (changing the transition field) in 2L CrI_3 [4], different from the emergent FM loop in 2L CrI_3/FL $CrCl_3$ heterostructure.

Therefore, it is unlikely that the bubble-induced strain plays an important role in our work.

(**c**) *Surface-related magnetism.* The surface-related magnetism may modulate the magnetic order. For example, it is reported that FM-like magnetic loops are observed in all even-number septuple-layered (SL) $MnBi_2Te_4$— including 2L $MnBi_2Te_4$— which is attributed to the "surface-related magnetism" of unknown origin [7]. In contrast, first we note that, in 2L CrI_3 region with the same top surface as 2L CrI_3/FL $CrCl_3$, we did not observe the emergent FM loop. This strongly suggests that our observations originate from the interfacial $CrCl_3$. We also studied reversely stacked heterostructures with FL $CrCl_3$ on top of 2L CrI_3 and observed similar FM loops (Supplementary Fig. 2) (while such FM loops are again absent in the exposed 2L CrI_3 region or FL $CrCl_3$ region on the same sample), which further lends support to the role of interfacial $CrCl_3$ proximal to CrI_3.

When forming an interface with $CrCl_3$, CrI_3 may be affected by the adjacent $CrCl_3$. However, it is well-established that the magnetic state in $CrCl_3$ is more susceptible as opposed to that in CrI_3. This is because $CrCl_3$ is located close to the boundary between perpendicular magnetic anisotropy (PMA) and in-plane anisotropy [8, 9]. Consistent with this, based on the DFT calculations presented in our original manuscript, we find that the interfacial FM exchange coupling in the heterostructure wins over the in-plane anisotropy of $CrCl_3$ and results in the out-of-plane magnetic order in the $CrCl_3$ layer next to CrI_3, in agreement with our observations.

Furthermore, we also do not believe disorders or surface impurities-related magnetism is the likely scenario because they usually lower the critical temperatures (T_C) [10, 11]. While for the heterostructure, we observed enhanced T_C. As shown in Fig. 2 and Supplementary Fig. 5, T_C of the FM-like hysteresis loop in the 2L CrI_3/FL $CrCl_3$ heterostructure (~48 K) is higher than that of either 2L CrI_3 (~40 K) and FL $CrCl_3$ (~18.6 K). Another experiment (Supplementary Fig. 4) on 1L CrI_3/FL $CrCl_3$ heterostructure shows T_C of ~ 33 K and ~ 37 K for 1L CrI_3 and the 1L CrI_3/FL $CrCl_3$ heterostructure, respectively. The enhanced T_C can be understood by the effective anisotropy field deriving from the interfacial ferromagnetic coupling in the heterostructure, which is expected to enlarge the spin-wave gaps for the adjacent magnetic materials [12-14].

Overall, the surface-related magnetism is thus also less likely to be an important mechanism explaining the experimental observations in our samples.

2. First-principles perspectives of the CrI_3/$CrCl_3$ heterostructures

DFT simulation is widely implemented to predict the magnetic properties of both monolayers [15, 16] and homobilayers [17, 18]. In this section, we demonstrate the results from our DFT calculations using the Vienna *ab-initio* Simulation Package (VASP) [19] to support the observed out-of-plane magnetic order as well as the interfacial ferromagnetic coupling in CrI_3/$CrCl_3$ heterostructures.

Homobilayer relaxation of CrI_3/CrI_3 and $CrCl_3$/$CrCl_3$ shows that their lattice constants are 6.82 Å and 5.93 Å respectively, consistent with the literature [18]. Therefore, we considered a supercell constructed by stacking $6\times6\times1$ CrI_3 supercell on top of $7\times7\times1$ $CrCl_3$ supercell (Supplementary Fig. 9), which forms a nearly-commensurate periodic unit (with small lattice mismatch $\frac{5.93Å\times7-6.82Å\times6}{5.93Å\times7}\times100\%\approx1.4\%$). As suggested in ref. [18], we employed PBEsol [20]

functional throughout our calculations. We also used a 3×3×1 Monkhorst-Pack k-point grid and chose the plane-wave energy cutoff of 300 eV [21]. We checked with other grid sizes and a higher energy cutoff of 400 eV, and the results indicate that our conclusion of interfacial ferromagnetic coupling is unaffected. Since the results of previous literature [22] show that magnetic configurations have little influence on the lattice structure of CrX_3 (X = I, Cl), we can relax the structure in one magnetic state and use the optimized structure throughout. Following ref. [18] and ref. [16], the structure was fully relaxed within a perpendicular ferromagnetic state (both intralayer and interlayer). The force convergence criterion was set to 30 meV/Å. In anisotropic magnetism calculations, we employed DFT+U method introduced by Liechtenstein et al. [23] to deal with strong correlations of Cr-*3d* electrons. We set effective on-site Coulomb interaction U=3 eV and Hund's rule coupling J_H=0 eV according to the benchmark calculations in a previous study [24]. Spin-orbit coupling (SOC) was included in all magnetic configuration calculations.

Twisting one CrI_3 layer relative to another layer with a small angle may lead to alternating stacking domains and non-collinear AFM-FM domains with sufficiently large moiré periodicity (typically $\gtrsim$ 10 nm for twisted CrI_3 [18, 25, 26]). For the CrI_3/$CrCl_3$ heterostructure, with a large lattice constant mismatch (6.82 Å and 5.93 Å for CrI_3 and $CrCl_3$, respectively [18]), our random stacking typically results in large twist angles and small nominal moiré periodicity (~4.5 nm for 0˚, ~1 nm for 30˚, calculated following Ref. [27]). For such small moiré periodicity, the energy cost of forming magnetic domain walls becomes too high that the system eventually collapses to a collinear phase with no domains [25, 26]. Therefore, instead of having moiré-related magnetic domains in the heterostructure, we consider an averaged magnetic moment in each magnetic layer.

We denote spins in each layer by a macroscopic spin (out-of-plane: ↑, ↓; in-plane: ←, →). In our calculations, we studied four types of magnetic configurations: perpendicular ferromagnetic state (↑↑), perpendicular antiferromagnetic state (↑↓), the state that CrI_3 is out-of-plane polarized while $CrCl_3$ in-plane polarized (↑→), and the state that CrI_3 is in-plane polarized while $CrCl_3$ out-of-plane polarized (→↑). The ground-state energies of the supercells are denoted as E_{FM}, E_{AFM}, $E_{\mathrm{CrI3out}}^{\mathrm{CrCl3in}}$ and $E_{\mathrm{CrCl3out}}^{\mathrm{CrI3in}}$, respectively. The self-consistent calculations show thatE_{FM}<$E_{\mathrm{CrI3out}}^{\mathrm{CrCl3in}}$<$E_{\mathrm{AFM}}$<$E_{\mathrm{CrCl3out}}^{\mathrm{CrI3in}}$, suggesting that bilayer perpendicular ferromagnetic state is the most stable one among the four configurations. This result supports the observed out-of-plane magnetic order and ferromagnetic coupling in CrI_3/$CrCl_3$ heterostructures. The data for relative

ground-state energies of different magnetic configurations in a given supercell are listed in Supplementary Table 1.

It should be noticed that DFT calculations do not take into account the magnetic dipole-dipole interaction, which is crucial for the in-plane magnetic anisotropy of $CrCl_3$ [18]. Previous work on numerical calculation of dipole-dipole interaction shows that its contribution to anisotropy energy is no more than 63 μJ/m^2 [18]. In presence of a nearby PMA magnet (such as CrI_3), this value is expected to be further reduced. Since $\frac{E_{\mathrm{CrI3out}}^{\mathrm{CrCl3in}}-E_{\mathrm{FM}}}{A_{\mathrm{Supercell}}}$ ≈237 μJ/m^2 >63 μJ/m^2 ($A_{\mathrm{Supercell}}$=14.922 nm^2 is the area of the supercell), further consideration on dipole-dipole interaction will not undermine the validity of the argument of the perpendicular ferromagnetic state as the preferred ground state in $CrI_3/CrCl_3$.

In order to ascertain whether a relative twist angle between the layers affects the results or not, we further conducted a similar calculation for $CrI_3/CrCl_3$ with a commensurate twist angle of 30° (Supplementary Fig. 10). The structure was constructed by *Twister* Package [28]. The relative ground-state energies are listed in Supplementary Table 1. Our results suggest that the general conclusions remain consistent even with a relative twist angle, i.e., $E_{\mathrm{FM}}<E_{\mathrm{CrI3out}}^{\mathrm{CrCl3in}}<E_{\mathrm{AFM}}<E_{\mathrm{CrCl3out}}^{\mathrm{CrI3in}}$ and $\frac{E_{\mathrm{CrI3out}}^{\mathrm{CrCl3in}}-E_{\mathrm{FM}}}{A_{\mathrm{Supercell}}}$ ≈184 μJ/m^2 >63 μJ/m^2 still hold ($A_{\mathrm{Supercell}}$=4.873 nm^2 for 30°-twisted bilayer). Therefore, the change of twist angle from 0° to 30° does not influence our conclusion of interfacial ferromagnetic coupling in $CrI_3/CrCl_3$ heterostructures, even if the local stacking configurations are altered.

Next, we proceed to make a quantitative estimation of the induced parameters. To this end, within a continuum model of locally coupled spin densities, the magnetic energy of the system (normalized to per unit area of the supercell) can be written as [17, 18]

$$E_{\mathrm{total}}/A_{\mathrm{Supercell}} = J_{\mathrm{inter}}\mathbf{S}_1 \cdot \mathbf{S}_2 - K_1 S_{1z}^2 - K_2 S_{2z}^2 + E_{\mathrm{intra}}^{\mathrm{ex}}, \qquad \text{(S1)}$$

where J_{inter} is the interlayer exchange energy in $CrI_3/CrCl_3$, $|S_{1(2)}|$=3/2 is the spin for Cr atom [29], $K_{1(2)}$ is the effective anisotropy for CrI_3 ($CrCl_3$) layer, $E_{\mathrm{intra}}^{\mathrm{ex}}$ is the isotropic part of intralayer exchange energy. Thus, the magnetic energies obtained from DFT can be written as

$$E_{\mathrm{FM}}/A_{\mathrm{Supercell}} = +J_{\mathrm{inter}} S_1 S_2 - K_1 S_1^2 - K_2 S_2^2 + E_{\mathrm{intra}}^{\mathrm{ex}}, \qquad \text{(S2)}$$

$$E_{\mathrm{AFM}}/A_{\mathrm{Supercell}} = -J_{\mathrm{inter}}S_1S_2 - K_1S_1^2 - K_2S_2^2 + E_{\mathrm{intra}}^{\mathrm{ex}}, \qquad \text{(S3)}$$

$$E_{\mathrm{CrI3out}}^{\mathrm{CrCl3in}}/A_{\mathrm{Supercell}} = -K_1S_1^2 + E_{\mathrm{intra}}^{\mathrm{ex}}, \qquad \text{(S4)}$$

$$E_{\mathrm{CrCl3out}}^{\mathrm{CrI3in}}/A_{\mathrm{Supercell}} = -K_2S_2^2 + E_{\mathrm{intra}}^{\mathrm{ex}}. \qquad \text{(S5)}$$

By the relative energies obtained in the DFT calculation in Supplementary Table 1, we can estimate ferromagnetic interlayer exchange energy between CrI_3 and $CrCl_3$ as $J_{\mathrm{inter}} = \frac{E_{\mathrm{FM}}-E_{\mathrm{AFM}}}{A_{\mathrm{Supercell}}\cdot 2S_1S_2} \approx$-77 μJ/m^2@0˚, -64 μJ/m^2@30˚ (for the two twist angle cases simulated), compared with the antiferromagnetic interlayer exchange in bilayer CrI_3, which is reported to be ~80 μJ/m^2 [13, 29, 30]. We can also estimate the effective anisotropy of CrI_3 in the heterostructure $K_1 = \frac{2E_{\mathrm{CrCl3out}}^{\mathrm{CrI3in}}-(E_{\mathrm{AFM}}+E_{\mathrm{FM}})}{A_{\mathrm{Supercell}}\cdot 2S_1^2} \approx$293 μJ/m^2@0˚, 287 μJ/m^2@30˚, larger than that of the intrinsic CrI_3 ~108 μJ/m^2 [29]. Such higher anisotropy of CrI_3 qualitatively agrees with the observed enhanced critical temperature ($T_C^*>T_C$ in Fig. 2).

The effective anisotropy of $CrCl_3$ in the heterostructure $K_2 = \frac{2E_{\mathrm{CrI3out}}^{\mathrm{CrCl3in}}-(E_{\mathrm{AFM}}+E_{\mathrm{FM}})}{S_{\mathrm{Supercell}}\cdot 2S_2^2} \approx 29$ μJ/m^2@0˚, 18 μJ/m^2@30˚. While the magnetic dipole-dipole interaction has little influence on the anisotropy of CrI_3 (K_1), it cannot be neglected for the weak anisotropy of $CrCl_3$ (K_2). The dipole-dipole interaction is no more than 63 μJ/m^2 in $CrCl_3$ as mentioned above and we can obtain the corrected $K_2 \approx$ -35 μJ/m^2@0˚, -45 μJ/m^2@30˚. $|J_{\mathrm{inter}}| > |K_2|$suggests that the interfacial exchange coupling in the $CrI_3/CrCl_3$ heterostructure wins over the in-plane anisotropy of $CrCl_3$ and results in the out-of-plane magnetic order in $CrCl_3$, as we observed in this work.

3. Analysis of the electric field tunability in $CrI_3/CrCl_3$ heterostructure

Electrical control of vdW magnets has garnered significant recent interest for advancing the fundamental understanding of magnetic phenomena and phase transitions in the atomic limit [5, 6, 31], as well as opened up opportunities for creating low-dissipation spintronic devices benefitting from vdW heterostructures [4, 5]. The coupling between spin and charge degrees of freedom in vdW heterostructures can result from charge doping-induced or/and electric field-induced modifications of magnetic interactions [5, 6, 31]. Consequently, the spin-charge coupling in such structures has been restricted to doping-induced modifications of the magnetic interactions [6, 31] (e.g., anisotropy and

interlayer exchange). On the other hand, electric fields can couple to the antiferromagnetic order in the presence of external magnetic fields (the so-called "magnetoelectric effect") [5].

To understand the electric field tunability of the $CrI_3/CrCl_3$ heterostructure, we consider here a dual gated $CrI_3/CrCl_3$ system with σ_1 and σ_2 charges introduced to the corresponding layers via capacitive gates. Similar to Equation (S1), the free energy per unit area of such a system under the macroscopic approximation can be written as

$$E/A = J_{\text{inter}}|S|^2\mathbf{m}_1 \cdot \mathbf{m}_2 - K_1|S|^2 m_{1z}^2 - K_2|S|^2 m_{2z}^2, \tag{S6}$$

where $\mathbf{m}_i$ and K_i are the magnetization and anisotropy of the respective layers, $|S_{1(2)}|=3/2$ is the spin for Cr atom, and J_{inter} is the interlayer exchange energy as described in the previous section. The charges couple to the magnetic order of the CrI_3 and $CrCl_3$ layers and the effect can be captured by introducing additional free energy terms in Equation (S6). The functional forms of such terms (E_{elec}) are dictated by the symmetries present in the system. In systems consisting of similar materials (such as 2L CrI_3), there exists a structural inversion symmetry that forces the free energy functional to remain invariant under the inversion transformation. Thus, under structural inversion $\mathbf{m}_1 \cdot \mathbf{m}_2$, m_{1z}^2 and m_{2z}^2 remain invariant, while $(\sigma_1 - \sigma_2)$ changes sign. Consequently, spin-charge coupling terms such as $(\sigma_1 + \sigma_2)\mathbf{m}_1 \cdot \mathbf{m}_2$, $(\sigma_1 + \sigma_2)m_{1z}^2$ and $(\sigma_1 + \sigma_2)m_{2z}^2$ are allowed whereas $(\sigma_1 - \sigma_2)\mathbf{m}_1 \cdot \mathbf{m}_2$, $(\sigma_1 - \sigma_2)m_{1z}^2$ and $(\sigma_1 - \sigma_2)m_{2z}^2$ are forbidden by symmetry. As a result, in hybrids with structural inversion, $(\sigma_1 + \sigma_2) \sim$ electrostatic doping can modulate interlayer exchange and anisotropy, while the antisymmetric combination $(\sigma_1 - \sigma_2) \sim$ electric field does not directly couple to those parameters. This is consistent with earlier reports on symmetric structures showing the electrical control of magnetic order achieved via electrostatic doping as opposed to electric-field-dominated effects [6, 31, 32]. The electric field can however couple with the Néel order, i.e., $(\mathbf{m}_1 - \mathbf{m}_2)$, in the presence of an external magnetic field $\mathbf{H}$ via the symmetry allowed magnetoelectric coupling term $\sim (\sigma_1 - \sigma_2)(\mathbf{m}_1 - \mathbf{m}_2) \cdot \mathbf{H}$ [5, 33].

According to the Neumann's principle [34], any physical property of the system must respect the system's inherent symmetries. We apply this principle to write down the spin-charge coupling Hamiltonian for heterobilayer system studied here (corresponding to $CrCl_3/CrI_3$) and compare it with the case of homobilayer of CrI_3. Most importantly, the heterobilayer (homobilayer) crystal breaks (respects) the spatial inversion symmetry I [2], which gives rise to spin-charge coupling terms in heterobilayers consistent with the experimental observations, as shown below. Moreover, we

exploit the presence of time-reversal symmetry (i.e., when magnetization is allowed to transform) and assume continuous spin rotations (C_θ) about the z-axis (oriented along the normal to the 2D magnet plane) as an additional symmetry to restrict the form of spin-charge coupling terms. We remark here that assuming C_θ amounts to neglecting the presence of a special crystal axis in the xy plane of the system. In principle, this assumption can be relaxed to write more general terms, such as charge-controlled in-plane magnetic anisotropies. However, in this work we do not focus on such terms for the following reasons: (i) in-plane magnetic anisotropies, even in pristine chromium halides of interest here, have been found to be parametrically smaller than out-of-plane anisotropies[16, 29, 35]; we thus expect the charge control of out-of-plane anisotropies to play a more dominant role, (ii) the spin-charge coupling we study here is observed in randomly stacked large-angle twisted structures, which further suggests the in-plane crystal structure does not play a major role in governing them, and (iii) the central experimental observation we would like the theory to explain is the charge-induced change in out-of-plane coercivities present in the heterobilayer, which is absent in the homobilayer system. Since the out-of-plane coercivities are dictated by the out-of-plane anisotropy, we focus on keeping such terms. To observe any effect within xy-plane magnetic anisotropy and its charge control, measurements sensitive to the in-plane magnetization component need to be performed, which is outside the scope of the present polar MOKE study.

The Hamiltonian of the homo- and heterobilayer systems can be expanded in terms of the magnetizations ($\mathbf{m}_i$), and charge densities (σ_i) of each layer as:

$$H[\mathbf{m}_1, \mathbf{m}_2, \sigma_1, \sigma_2] = H^{(1)} + H^{(2)} + \cdots. \quad \text{(S7)}$$

Here, in the spirit of linear response, we only keep terms which are linear in σ_i, while including terms up to the second order in $\mathbf{m}_i$ (to capture magnetic anisotropies and interlayer exchange); the superscript on the right-hand side indicates the order of the term with respect to $\mathbf{m}_i$. The zeroth-order terms correspond to a constant reference and thus do not contribute to spin-charge coupling. Next, we will enumerate possible first- and second-order terms and only keep the ones that respect the symmetries of the system to arrive at the spin-charge coupling Hamiltonian.

The first-order spin-charge coupling terms can be written as:

$$H^{(1)} = \sum_{i=x,y,z} \lambda_{i1,\pm} \sigma_\pm m_{i1} + \sum_{i=x,y,z} \lambda_{i2,\pm} \sigma_\pm m_{i2}, \quad \text{(S8)}$$

where $\sigma_+ = (\sigma_1 + \sigma_2)$ and $\sigma_- = (\sigma_1 - \sigma_2)$ are proportional to the electrostatic doping and electric field applied to the system respectively, and $\lambda_{i1,\pm}, \lambda_{i2,\pm}$ are the strength of such interactions. Since, under time reversal: $\mathbf{m} \rightarrow -\mathbf{m}$, and $\sigma_\pm \rightarrow \sigma_\pm$, the above-mentioned spin-charge coupling terms become forbidden for both the homo- and heterobilayer systems.

For the second-order terms, we can write them as:

$$H^{(2)} = \sum_{i=x,y,z} \lambda'_{i1,\pm}\sigma_\pm m_{i1}^2 + \sum_{i=x,y,z} \lambda'_{i2,\pm}\sigma_\pm m_{i2}^2 + \sum_{i,j=x,y,z} \lambda'_{ij,\pm}\sigma_\pm m_{i1} m_{j2}. \quad \text{(S9)}$$

For homobilayers, I symmetry (under which $\sigma_- \rightarrow -\sigma_-$) prevents any electric field-induced terms to be present in the expansion. However, for heterobilayers, breaking of I allows the electric field-induced terms. In both cases, a symmetric combination of the charges, i.e., electrostatic doping $\sim \sigma_+$ (which remains invariant under I) can nonetheless couple. In addition, upon invoking C_θ, we can simplify Eq. (S9) to keep terms that (i) depend exclusively on the out-of-plane magnetic components [i.e., out-of-plane anisotropy type terms proportional to m_{z1}^2, m_{z2}^2 from the first two terms on the right-hand side on Eq. (S9)], and (ii) depend only on the relative angle between $\mathbf{m}_1$ and $\mathbf{m}_2$ [i.e. interlayer exchange type terms from the last term on the right-hand side on Eq. (S9)]. Thus, the electric-field induced spin-charge coupling terms that are only allowed in heterobilayers (but not in homobilayers) can be succinctly written as the electric-field-dependent perpendicular anisotropy and interlayer exchange (which is the equation used in the main manuscript):

$$H^{(2)} = \sigma_-(\,\beta_1 m_{z1}^2 + \beta_2 m_{z2}^2 + \beta_3\, \mathbf{m}_1 \cdot \mathbf{m}_2). \quad \text{(S10)}$$

Here, for simplifying notations, we have redefined the variables: $\beta_1 = \lambda'_{z1,-}$, $\beta_2 = \lambda'_{z2,-}$, and $\beta_3 = \lambda'_{xx,-} = \lambda'_{yy,-} = \lambda'_{zz,-}$ (we note that in principle $\lambda'_{xx,-} = \lambda'_{yy,-} \neq \lambda'_{zz,-}$ is allowed by the symmetries considered here, but in line with experiments for pristine chromium halides, we have neglected such anisotropic interlayer exchange coupling).

Therefore, breaking the structural inversion symmetry by choosing two different layers for the heterostructure allows for spin-charge coupling terms which can couple directly to electric fields. These terms can be written as

$$E_{\text{elec}}(\mathbf{m}_i, \sigma_i) = (\sigma_1 - \sigma_2)(\beta_1 m_{z1}^2 + \beta_2 m_{z2}^2 + \beta_3 \mathbf{m}_1 \cdot \mathbf{m}_2), \quad \text{(S11)}$$

and can be understood as electric field control of magnetic anisotropy (ECMA) and interlayer exchange (ECIJ), respectively, where $\beta_{1,2,3}$ parameterizes the strength of such interactions. In

particular, since the coercivity is predominantly controlled by magnetic anisotropy, the observed electric field control of coercivity in Fig. 3c,e seems consistent with the predicted ECMA terms.

4. Electric control of the magnetism in FL $CrCl_3$/2L CrI_3 heterostructure

The intriguing electrical tunability allowed by symmetry breaking is also observed in a heterostructure containing a bilayer CrI_3. Supplementary Fig. 11a,b shows the optical micrograph and schematic structure of a reversely stacked FL $CrCl_3$/2L CrI_3 device. Supplementary Fig. 11c,d presents θ_K as a function of both V_{bg} and perpendicular magnetic field in 2L CrI_3 and the heterostructure, respectively (see representative MOKE curves in Supplementary Fig. 3). Similar to the heterostructure in Fig. 1, we also observed the antiferromagnetic spin-flip transitions in 2L CrI_3 and the ferromagnetic coercivity in the FL $CrCl_3$/2L CrI_3 heterostructure (dashed lines in Supplementary Fig. 11d). In 2L CrI_3, the critical field of antiferromagnetic spin-flip shrinks with increasing V_{bg}. This observation is consistent with the previous reports [6, 31] and derives from the effective tuning of the interlayer exchange interaction by electrostatic doping effect instead of the displacement field.

In the heterostructure, the electrical control of critical field of the antiferromagnetic spin-flip weakens, presumably due to the doped-charge redistribution to the contacting $CrCl_3$ layer. Remarkably, the interfacial ferromagnetism is highly controllable by electrostatic gating. The coercivity significantly shrinks with increasing V_{bg} as shown by the dashed lines. Such modulation to the interfacial ferromagnetism in 2L CrI_3/$CrCl_3$ heterostructure is similar to the monolayer case discussed in the main text, where the structural inversion symmetry breaking in the heterostructure allows for spin-charge coupling terms which can couple to the electric field.

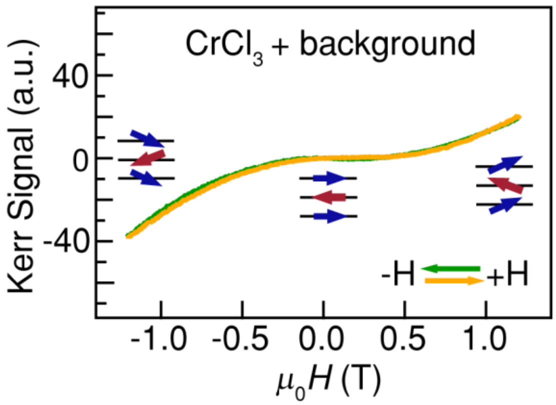

Supplementary Fig. 1 The MOKE signal in the FL $CrCl_3$ region. Due to the spin-canting effect under perpendicular magnetic field, the $CrCl_3$ undergoes a continuous spin-flop transition from in-plane to fully out-of-plane spin alignment under perpendicular magnetic field larger than 2.4 T (Supplementary Fig. 5). In the mainly studied magnetic field range ~1 T, the MOKE signal of $CrCl_3$ is mixed with the continuously changing background signal, e.g., Faraday effect of optical components under magnetic field. By subtracting a polynomial background, both the background signal and the spin-flop MOKE signal of $CrCl_3$ are eliminated in all the MOKE curves measured on regions containing $CrCl_3$.

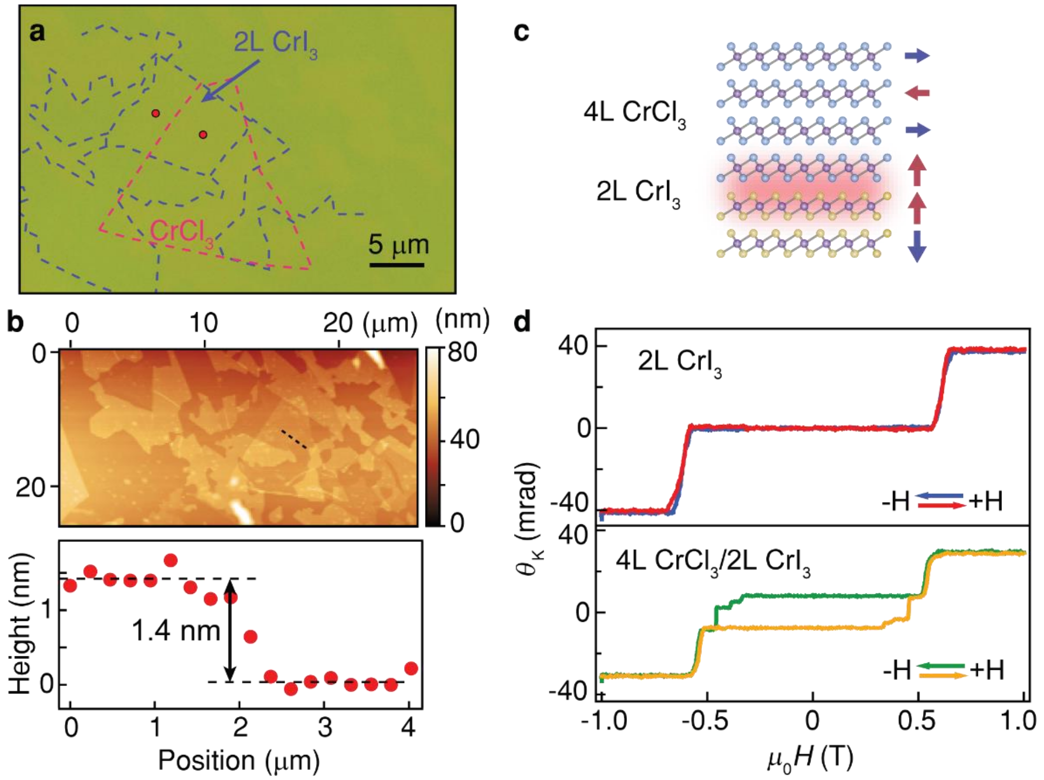

Supplementary Fig. 2 MOKE measurements on the heterostructure formed with a four-layer (4L) $CrCl_3$ on top of a 2L CrI_3. a, Optical micrograph of the 4L $CrCl_3$/2L CrI_3 heterostructure. The stack is encapsulated by two hBN flakes. **b,** AFM of the heterostructure in the same position as in **a**. Profile at the edge of CrI_3 indicates the bilayer thickness. **c,** Magnetic ground states of the 4L $CrCl_3$/2L CrI_3 heterostructure. Cr^{3+}, I^- and Cl^- ions are shown as purple, yellow and aqua blue balls, respectively. **d,** MOKE signal of 2L CrI_3 and 4L $CrCl_3$/2L CrI_3 heterostructure as a function of perpendicular magnetic field. The data is taken at the spots marked by red in **a**.

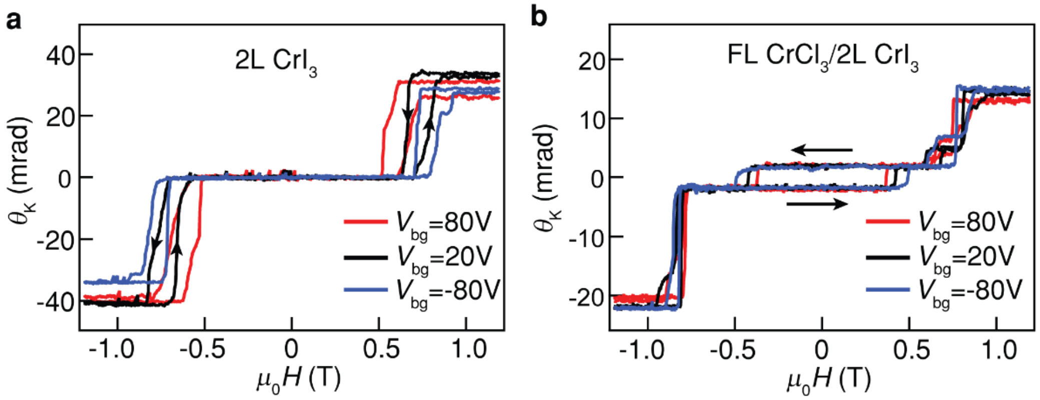

Supplementary Fig. 3 Electrical control of the magnetism in FL $CrCl_3$/2L CrI_3 heterostructure. MOKE signal as a function of perpendicular magnetic field in 2L CrI_3 region (**a**) and FL $CrCl_3$/2L CrI_3 heterostructure region (**b**) (from the same CrI_3 flake) at three representative back-gate voltages for the device shown in Supplementary Fig. 11. Two curves of each voltage represent forward and backward sweeps of the field, respectively.

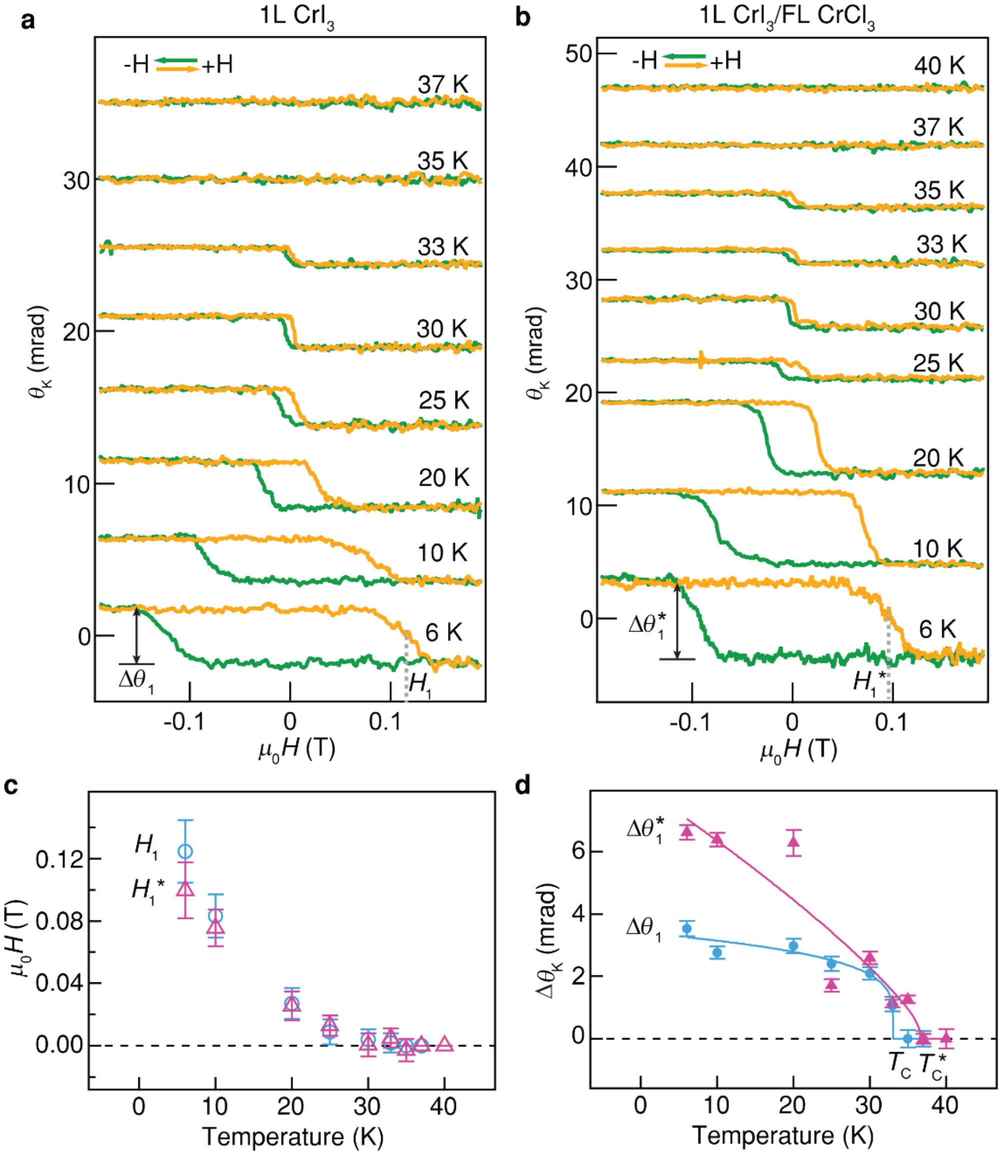

Supplementary Fig. 4 Temperature dependence of the magnetism of the 1L CrI_3 and the 1L CrI_3/FL $CrCl_3$ heterostructure. a,b, MOKE signal in (A) the 1L CrI_3 region and (B) the 1L CrI_3/FL $CrCl_3$ heterostructure region as a function of perpendicular magnetic field at different temperatures (from the same CrI_3 flake as in the heterostructure region). Coercive fields H_1, H_1^* and magnitudes in the change of MOKE signal $\Delta\theta_1$, $\Delta\theta_1^*$ of the magnetic transitions are labeled. **c,** Temperature dependence of the coercive fields H_1, H_1^*, extracted from the MOKE signal. The error bars correspond to the peak width of the derivative $d\theta_K/dH$ at the transitions. **d,** Magnitudes in the change of MOKE signal $\Delta\theta_1$, $\Delta\theta_1^*$ as a function of temperature. The power-law fitting curves are the guide to the eyes. The critical temperatures $T_C \sim 33$ K and $T_C^* \sim 37$ K are indicated. The error bars are the uncertainties in extracting the transition magnitudes.

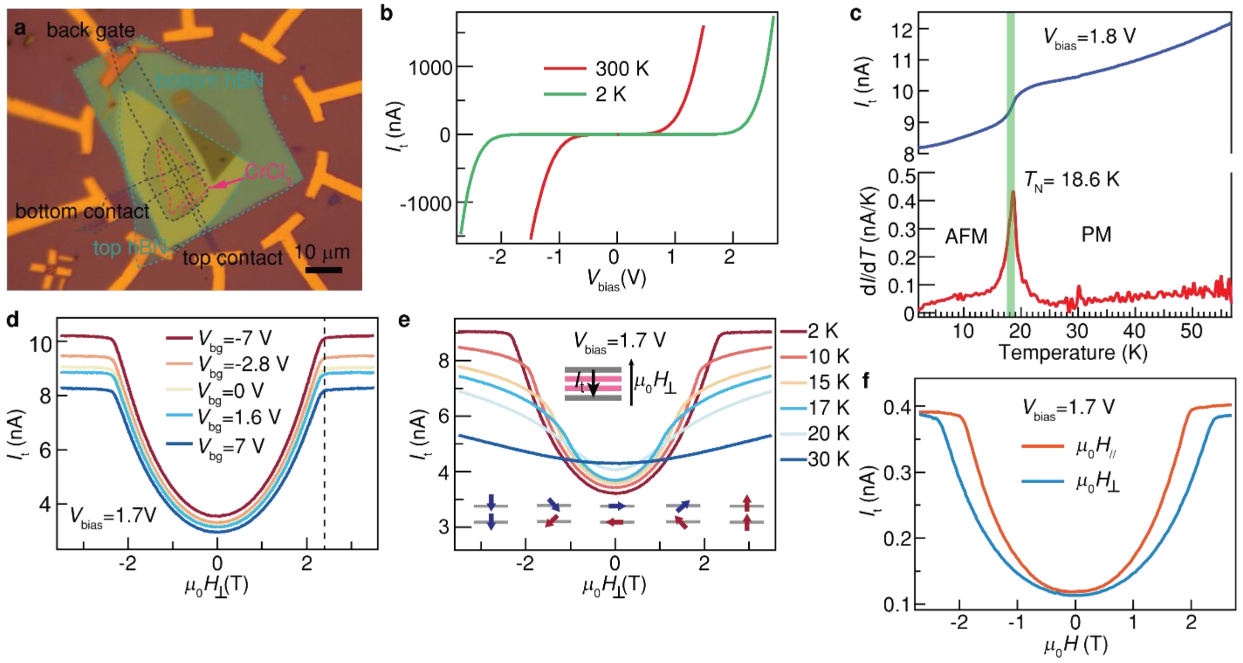

Supplementary Fig. 5 Transport measurements of spin-filtering magnetic tunneling junctions graphene/$CrCl_3$/graphene. a, Optical micrograph of a $CrCl_3$ device. The thickness of $CrCl_3$ is 3.8 nm. Three few-layer graphene flakes are used as back gate and bottom/top contact to $CrCl_3$. **b,** Tunneling current as a function of dc bias voltage taken at 300 K and 2 K, respectively. **c,** Tunneling current and the derivative of tunneling current as a function of temperature at zero magnetic field with dc bias voltage of 1.8 V. Green line indicates the Neel temperature of 18.6 K. **d,** Tunneling current as a function of perpendicular magnetic field with dc bias voltage of 1.7 V at different back-gate voltages of -7 V, -2.8 V, 0 V, 1.6 V, 7 V. Dash line denotes the critical field corresponding to saturation magnetization. Bilayer graphene is used as bottom contact so that the gating field is not fully screened [31] and can effectively dope charges to $CrCl_3$. **e,** Tunneling current as a function of perpendicular magnetic field with dc bias voltage of 1.7 V at temperatures of 2 K, 10 K, 15 K, 17 K, 20 K, 30 K, respectively. **f,** Another $CrCl_3$ device with a thickness of 6.1 nm, showing the comparison of tunneling current under in-plane and out-of-plane magnetic fields, consistent with the in-plane anisotropy of $CrCl_3$. Data are obtained at 2 K.

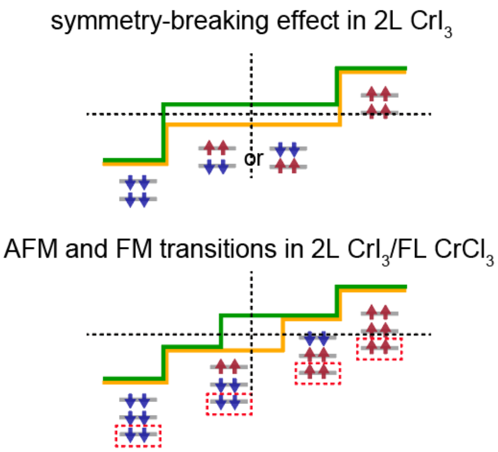

Supplementary Fig. 6 Comparison with symmetry-breaking effect. Schematics of the MOKE signal as a function of magnetic field for symmetry-breaking effect in 2L CrI_3 (upper panel) (supported by the reported electric-field-controlled magnetism in 2L CrI_3 [5]) and the coexistence of AFM-type and FM-type transitions in 2L CrI_3/FL $CrCl_3$ heterostructures (lower panel, corresponding to our experiment).

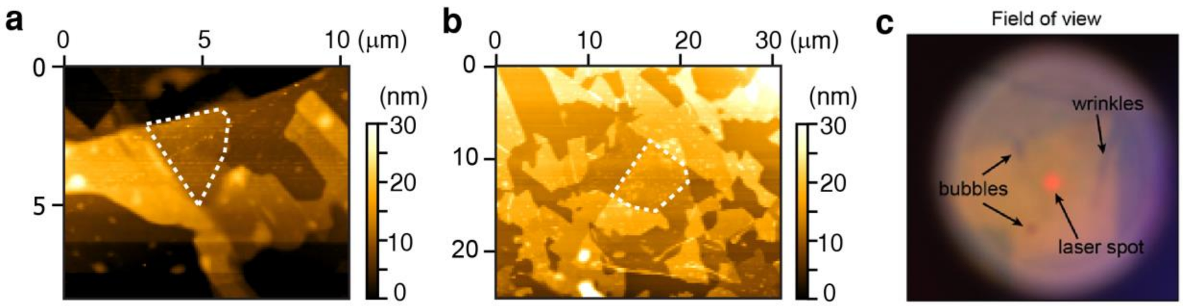

Supplementary Fig. 7 The uniform heterostructure interfaces with few bubbles. a,b, Typical AFM images of the 2L CrI_3/FL $CrCl_3$ heterostructures. The heterostructure regions are outlined by white dashed lines. **c,** The field of view of a typical sample under the microscope of the MOKE setup. The visible bubbles, wrinkles and laser spot are indicated by arrows.

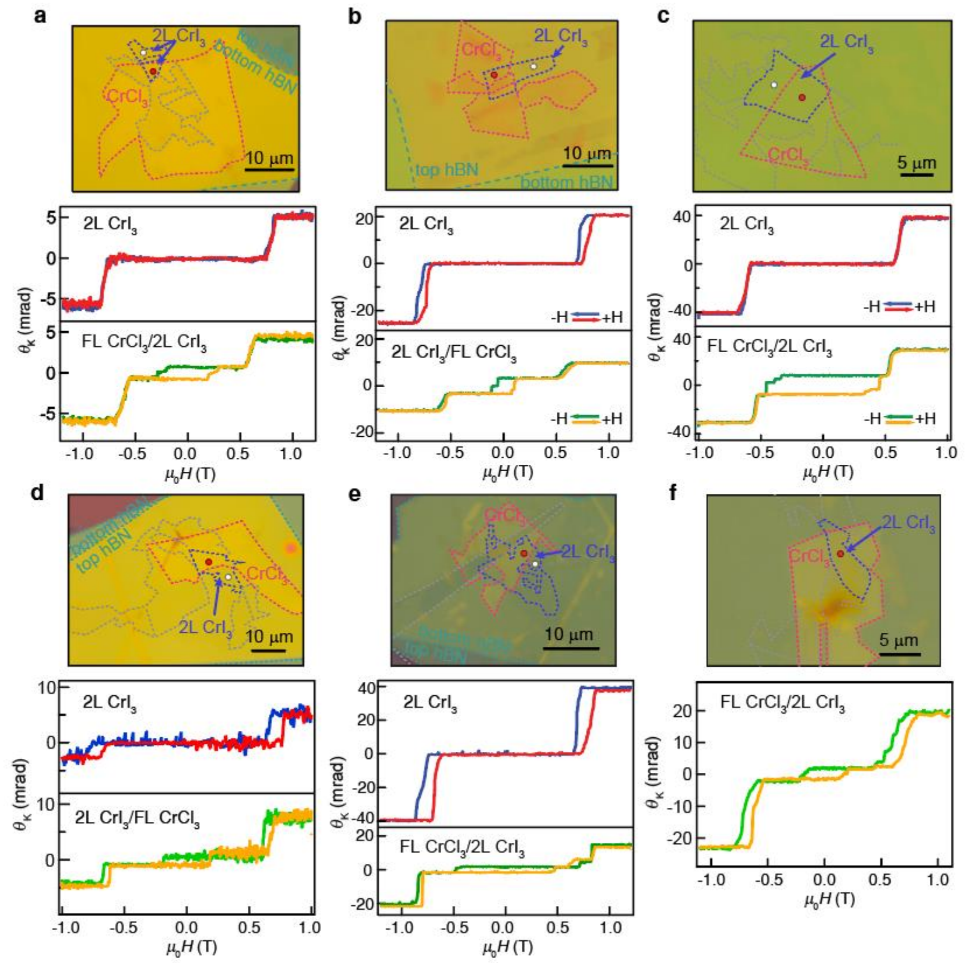

Supplementary Fig. 8 MOKE results in bilayer CrI_3 and 2L CrI_3/FL $CrCl_3$ or the reversed stack FL $CrCl_3$/2L CrI_3 of different samples. The optical micrograph on top of each MOKE result shows the 2L CrI_3 and FL $CrCl_3$, outlined by the blue and red dashed lines, respectively. Thicker flakes or other materials are outlined by gray dashed lines. The MOKE signals in 2L CrI_3 and the heterostructure (based on the same 2L CrI_3 flake) are obtained at the white and red spots denoted in each sample image.

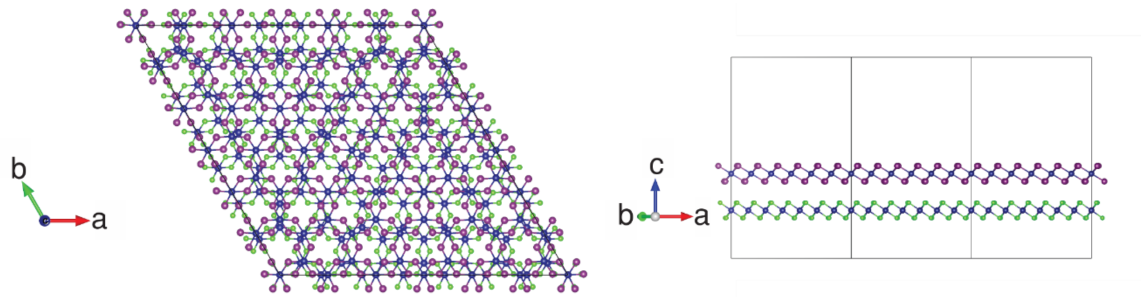

Supplementary Fig. 9 Top view (left) and side view (right) of supercell of 6×6×1 CrI_3/7×7×1 $CrCl_3$ bilayer with zero twist angle. Blue, purple and green balls represent Cr, I and Cl atoms, respectively. (Plot using VESTA [36])

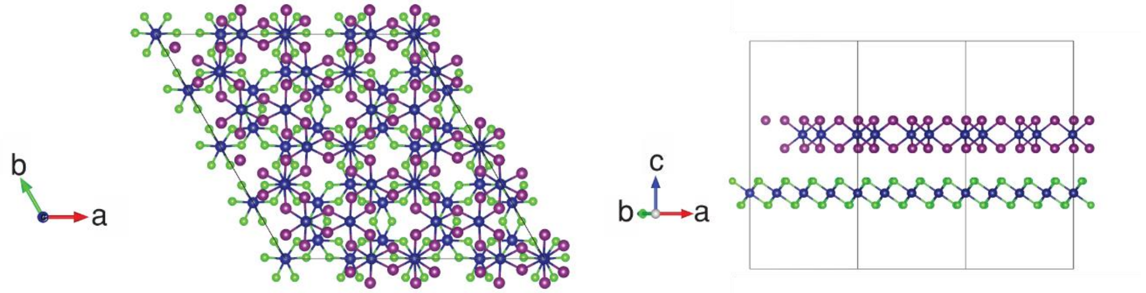

Supplementary Fig. 10 Top view (left) and side view (right) of supercell of 30°-twisted CrI_3/$CrCl_3$ bilayer. Blue, purple and green balls represent Cr, I and Cl atoms, respectively. (Plot using VESTA [36])

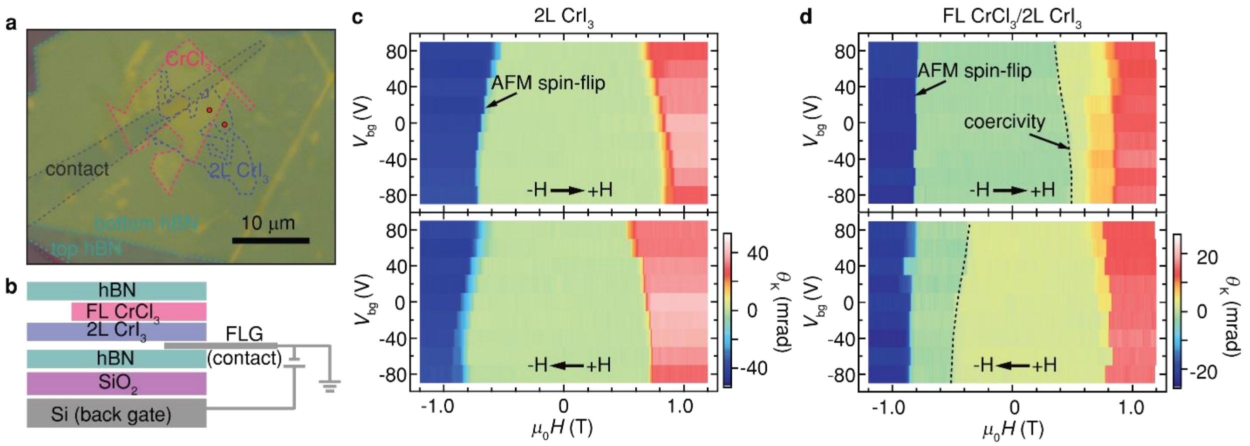

Supplementary Fig. 11 Electrical control of the magnetism in FL $CrCl_3$/2L CrI_3 heterostructure. a,b, Optical micrograph and schematic structure of a back-gated and reversely stacked FL $CrCl_3$/2L CrI_3 device. **c,d,** MOKE signal as a function of both back-gate voltage V_{bg} and perpendicular magnetic field in 2L CrI_3 (**c**) and FL $CrCl_3$/2L CrI_3 heterostructure (**d**). The top and bottom panels correspond to the forward and backward sweeps of the field, respectively. The dashed lines in **d** are the guide to the eyes. The data is taken at the spots marked by red in **a**.

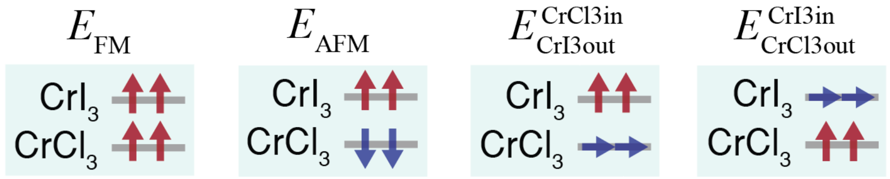

Relative supercell energy (meV)	$E_{AFM} - E_{FM}$	$E^{CrCl3in}_{CrI3out} - E_{FM}$	$E^{CrI3in}_{CrCl3out} - E_{FM}$
0°-twisted $CrI_3/CrCl_3$ bilayer	32.11	22.03	77.44
30°-twisted $CrI_3/CrCl_3$ bilayer	8.77	5.61	24.03

Supplementary Table 1. Relative ground-state energies for different stacking and magnetic configurations. The spin configurations for each magnetic state are schematically illustrated above the table.

Supplementary References

1. Sivadas, N., Okamoto, S. & Xiao, D. Gate-controllable magneto-optic Kerr effect in layered collinear antiferromagnets. *Phys. Rev. Lett.* **117**, 267203 (2016).
2. Yang, K., Hu, W., Wu, H., Whangbo, M.-H., Radaelli, P. G. & Stroppa, A. Magneto-optical Kerr switching properties of $(CrI_3)_2$ and $(CrBr_3/CrI_3)$ bilayers. *ACS Appl. Electron. Mater.* **2**, 1373-1380 (2020).
3. Lin, C.-L., *et al.* Substrate-induced symmetry breaking in silicene. *Phys. Rev. Lett.* **110**, 076801 (2013).
4. Jiang, S. W., Xie, H. C., Shan, J. & Mak, K. F. Exchange magnetostriction in two-dimensional antiferromagnets. *Nat. Mater.* **19**, 1295-1299 (2020).
5. Jiang, S. W., Shan, J. & Mak, K. F. Electric-field switching of two-dimensional van der Waals magnets. *Nat. Mater.* **17**, 406-410 (2018).
6. Huang, B., *et al.* Electrical control of 2D magnetism in bilayer CrI_3. *Nat. Nanotechnol.* **13**, 544-548 (2018).
7. Yang, S., *et al.* Odd-even layer-number effect and layer-dependent magnetic phase diagrams in $MnBi_2Te_4$. *Phys. Rev. X* **11**, 011003 (2021).
8. Tartaglia, T. A., *et al.* Accessing new magnetic regimes by tuning the ligand spin-orbit coupling in van der Waals magnets. *Sci. Adv.* **6**, eabb9379 (2020).
9. Wang, Z., *et al.* Determining the phase diagram of atomically thin layered antiferromagnet $CrCl_3$. *Nat. Nanotechnol.* **14**, 1116-1122 (2019).
10. Xu, J., Yang, Y., Xiong, H. & Lin, X. Monte Carlo simulation study of the influence of defects on two-dimensional ferromagnetic order. *AIP Adv.* **11**, 085016 (2021).
11. Salafranca, J. & Brey, L. Disorder-induced first order transition and Curie temperature lowering in ferromagnetic manganites. *Phys. Rev. B* **73**, 214404 (2006).
12. Gibertini, M., Koperski, M., Morpurgo, A. F. & Novoselov, K. S. Magnetic 2D materials and heterostructures. *Nat. Nanotechnol.* **14**, 408-419 (2019).
13. Lu, X., Fei, R. & Yang, L. Curie temperature of emerging two-dimensional magnetic structures. *Phys. Rev. B* **100**, 205409 (2019).
14. Gong, C., *et al.* Discovery of intrinsic ferromagnetism in two-dimensional van der Waals crystals. *Nature* **546**, 265-269 (2017).
15. Torelli, D., Moustafa, H., Jacobsen, K. W. & Olsen, T. High-throughput computational screening for two-dimensional magnetic materials based on experimental databases of three-dimensional compounds. *npj Comput. Mater.* **6**, 158 (2020).
16. Webster, L. & Yan, J.-A. Strain-tunable magnetic anisotropy in monolayer $CrCl_3$, $CrBr_3$, and CrI_3. *Phys. Rev. B* **98**, 144411 (2018).
17. Sivadas, N., Okamoto, S., Xu, X. D., Fennie, C. J. & Xiao, D. Stacking-dependent magnetism in bilayer CrI_3. *Nano Lett.* **18**, 7658-7664 (2018).
18. Akram, M., LaBollita, H., Dey, D., Kapeghian, J., Erten, O. & Botana, A. S. Moiré skyrmions and chiral magnetic phases in twisted CrX_3 (X = I, Br, and Cl) bilayers. *Nano Lett.* **21**, 6633-6639 (2021).
19. Kresse, G. & Furthmüller, J. Efficient iterative schemes for ab initio total-energy calculations using a plane-wave basis set. *Phys. Rev. B* **54**, 11169-11186 (1996).
20. Perdew, J. P., *et al.* Restoring the density-gradient expansion for exchange in solids and surfaces. *Phys. Rev. Lett.* **100**, 136406 (2008).
21. Larson, D. T. & Kaxiras, E. Raman spectrum of CrI_3: An ab initio study. *Phys. Rev. B* **98**, 085406 (2018).
22. Webster, L., Liang, L. & Yan, J.-A. Distinct spin–lattice and spin–phonon interactions in monolayer magnetic CrI_3. *Phys. Chem. Chem. Phys.* **20**, 23546-23555 (2018).
23. Liechtenstein, A. I., Anisimov, V. I. & Zaanen, J. Density-functional theory and strong interactions: Orbital ordering in Mott-Hubbard insulators. *Phys. Rev. B* **52**, R5467-R5470 (1995).

24. Lu, X., Fei, R., Zhu, L. & Yang, L. Meron-like topological spin defects in monolayer $CrCl_3$. *Nat. Commun.* **11**, 4724 (2020).
25. Xu, Y., *et al.* Coexisting ferromagnetic–antiferromagnetic state in twisted bilayer CrI_3. *Nat. Nanotechnol.* **17**, 143-147 (2021).
26. Hejazi, K., Luo, Z. X. & Balents, L. Noncollinear phases in moiré magnets. *Proc. Natl. Acad. Sci. U.S.A.* **117**, 10721-10726 (2020).
27. Yankowitz, M., *et al.* Emergence of superlattice Dirac points in graphene on hexagonal boron nitride. *Nat. Phys.* **8**, 382-386 (2012).
28. Naik, M. H. & Jain, M. Ultraflatbands and shear solitons in moiré patterns of twisted bilayer transition metal dichalcogenides. *Phys. Rev. Lett.* **121**, 266401 (2018).
29. Lado, J. L. & Fernández-Rossier, J. On the origin of magnetic anisotropy in two dimensional CrI_3. *2D Mater.* **4**, 035002 (2017).
30. Cenker, J., *et al.* Direct observation of two-dimensional magnons in atomically thin CrI_3. *Nat. Phys.* **17**, 20-25 (2021).
31. Jiang, S. W., Li, L. Z., Wang, Z. F., Mak, K. F. & Shan, J. Controlling magnetism in 2D CrI_3 by electrostatic doping. *Nat. Nanotechnol.* **13**, 549-553 (2018).
32. Huang, B., *et al.* Layer-dependent ferromagnetism in a van der Waals crystal down to the monolayer limit. *Nature* **546**, 270-273 (2017).
33. Rustagi, A., Solanki, A. B., Tserkovnyak, Y. & Upadhyaya, P. Coupled spin-charge dynamics in magnetic van der Waals heterostructures. *Phys. Rev. B* **102**, 094421 (2020).
34. Birss, R. R. *Symmetry and Magnetism* (North-Holland, Amsterdam, 1966).
35. Kim, J., *et al.* Exploitable magnetic anisotropy of the two-dimensional Magnet CrI_3. *Nano Lett.* **20**, 929-935 (2020).
36. Momma, K. & Izumi, F. VESTA 3 for three-dimensional visualization of crystal, volumetric and morphology data. *J. Appl. Crystallogr.* **44**, 1272-1276 (2011).
